# Structural, Molecular, and Functional Alterations of the Blood-Brain Barrier during Epileptogenesis and Epilepsy: A Cause, Consequence, or Both?

**DOI:** 10.3390/ijms21020591

**Published:** 2020-01-16

**Authors:** Wolfgang Löscher, Alon Friedman

**Affiliations:** 1 Department of Pharmacology, Toxicology and Pharmacy, University of Veterinary Medicine Hannover, 30559 Hannover, Germany; 2 Center of Systems Neuroscience, 30559 Hannover, Germany; 3 Departments of Physiology and Cell Biology, Brain and Cognitive Sciences, Zlowotski Center for Neuroscience, Ben-Gurion University of the Negev, Beer-Sheva 8410501, Israel; alonf@bgu.ac.il; 4 Department of Medical Neuroscience, Dalhousie University, Halifax, NS B3H 4R2, Canada

**Keywords:** tight junctions, antiepileptic drugs, epileptogenesis, albumin, P-glycoprotein

## Abstract

The blood-brain barrier (BBB) is a dynamic, highly selective barrier primarily formed by endothelial cells connected by tight junctions that separate the circulating blood from the brain extracellular fluid. The endothelial cells lining the brain microvessels are under the inductive influence of neighboring cell types, including astrocytes and pericytes. In addition to the anatomical characteristics of the BBB, various specific transport systems, enzymes and receptors regulate molecular and cellular traffic across the BBB. While the intact BBB prevents many macromolecules and immune cells from entering the brain, following epileptogenic brain insults the BBB changes its properties. Among BBB alterations, albumin extravasation and diapedesis of leucocytes from blood into brain parenchyma occur, inducing or contributing to epileptogenesis. Furthermore, seizures themselves may modulate BBB functions, permitting albumin extravasation, leading to activation of astrocytes and the innate immune system, and eventually modifications of neuronal networks. BBB alterations following seizures are not necessarily associated with enhanced drug penetration into the brain. Increased expression of multidrug efflux transporters such as P-glycoprotein likely act as a ‘second line defense’ mechanism to protect the brain from toxins. A better understanding of the complex alterations in BBB structure and function following seizures and in epilepsy may lead to novel therapeutic interventions allowing the prevention and treatment of epilepsy as well as other detrimental neuro-psychiatric sequelae of brain injury.

## 1. Introduction

The blood–brain barrier (BBB) is a dynamic and complex barrier essential for the normal function of the central nervous system (CNS). The existence of a physical interface between the CNS and the blood was first described by Paul Ehrlich in 1885 [1]. Ehrlich discovered that injection of a hydrophilic dye into the blood circulation stained peripheral organs but not the spinal cord and the brain. In 1921, Lisa Stern postulated a barrier between blood and neuronal tissue, coining the term blood–brain barrier (barrière hématoencéphalique) [2].

**Figure 1:**
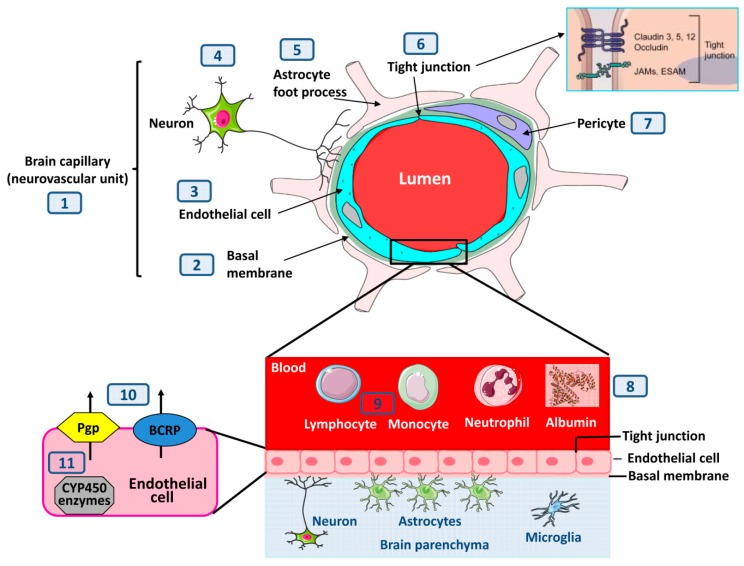
A schematic overview of the various morphological and biochemical constituents of the blood–brain barrier (BBB) and how they relate to alterations in response to seizures or epilepsy. As described in the text, seizures or processes underlying seizures may affect brain capillaries (also termed neurovascular unit (**1**)) by: thickening of the basal membrane (**2**), alterations of the endothelial cells that form the BBB (**3**), of neurons that are involved in the neurovascular unit (**4**), of astrocyte foot processes (**5**) that form a second barrier, termed the glia limitans, of tight junctions (**6**) between the endothelial cells, and of pericytes (**7**). The inset at (**6**) shows an enhanced schematic view of the molecular composition of endothelial tight junctions; occludin and the claudin proteins are the most important membranous components of the tight junctions. Junctional adhesion molecules (JAMs) and endothelial selective adhesion molecule (ESAM) are members of an immunoglobulin superfamily involved in the formation and maintenance of tight junctions. In addition to directly affecting the different anatomical components of the BBB, epileptogenic brain injury typically leads to extravasation of albumin (**8**) and blood-born immune cells (**9**) into the brain parenchyma. Possibly as a ‘second line of defense’ mechanism, BBB disruption is associated with increased expression of efflux transporters such as Pgp and BCRP at the apical site of BBB endothelial cells (**10**) and with increased activity of drug-metabolizing CYP450 enzymes in the endothelial cells (**11**).

The BBB plays a crucial role in maintaining strict homeostasis of the neuropil extracellular environment [3,4,5,6]. The BBB is primarily formed by endothelial cells lining the brain microvessels (Figure 1). Tight junctions between endothelial cells limit the paracellular flux of hydrophilic and macromolecules across the BBB, while nutrients including glucose and amino acids enter the brain via specific membrane transporters. As shown in Figure 1, tight junctions are a type of cell–cell barrier formed by a complex of proteins that span the intercellular cleft–occludin, claudins, junctional adhesion molecules (JAMs), as well as endothelial selective adhesion molecule (ESAM) [4].

In addition to endothelial cells, the BBB is composed of the capillary basal or basement membrane, comprised of collagen type 4, elastin, fibrillin, laminin, fibronectin and extracellular matrix proteins, and pericytes embedded within the basal membrane (Figure 1). Importantly, the glia limitans, formed by astrocytic end-feet processes that surround the endothelial cells, add to the barrier properties. There has been a long-standing interest in how the brain regulates its own blood supply, specifically in the vascular response to neuronal activity. In this context, the concept of the neurovascular unit (NVU) emerged to emphasize the unique neuro-vascular interactions that control cerebral blood flow and other functions of the brain blood vessels [7].

The BBB largely functions as a diffusion barrier for drugs (or xenobiotics) limiting drug penetration through the BBB to small (<500 Da), lipophilic and uncharged compounds [4]. Additional physicochemical parameters of drugs that affect BBB permeability include hydrogen bond donors and acceptors and the polar surface area of the drugs [8]. Consequently, >98% of all small molecule drugs do not cross the BBB [9]. Antiseizure drugs (ASDs; previously termed antiepileptic drugs) are typically small, lipophilic and uncharged and can thus easily access the CNS by passive diffusion [10]. In addition to passive diffusion, a few drugs may use carrier-mediated active transport at the BBB to penetrate the brain parenchyma [11]. One example is the short branched-chain fatty acid valproate, an ASD that is actively transported into and out of the CNS by probenecid-sensitive carriers [12], with brain uptake being mediated by monocarboxylate transporter 1 [13].

Notably, the NVU in general (and BBB in particular) is not uniform throughout the brain [14]. For example, at the level of circumventricular organs (such as the area postrema, posterior pituitary, intermediate lobe of the pituitary gland, median eminence, subcommissural organ, pineal gland, subfornical organ, and the organum vasculosum laminae terminalis) capillaries are more permeable, containing fenestrations and discontinuous tight junctions. Although various compounds, including xenobiotics, can enter these regions from the blood, this does not allow direct penetration of blood-borne substances to the rest of the brain due to the presence of diffusion barriers [14].

In addition to the structural barrier, particularly the tight junctions between endothelial cells, a number of ABC energy-dependent efflux transporters (ATP-binding cassette transporters), including P-glycoprotein (Pgp; MDR1; ABCB1) and breast cancer resistance protein (BCRP, ABCG2), provide a chemical barrier in brain capillary endothelial cells by actively pumping potentially toxic lipophilic compounds back into the blood, and restricting the brain entry of many therapeutically used drugs [15,16]. Furthermore, drug-metabolizing cytochrome (CYP)P450 enzymes are present in brain capillary endothelial cells and are thought to add to the chemical barrier of the BBB [17].

## 2. Seizures and the BBB

The first indication that epileptic seizures may compromise the functionality of the BBB stems from experiments in the 1950s, in which protein-bound dyes (Evans Blue or Geigy-Blau 536) were shown within the brain neuropil following pentylenetetrazole (PTZ)-induced seizures, but not in the control brain [18,19]. Subsequent studies using hydrophilic, small and large molecular weight tracers (e.g., horseradish peroxidase or sodium fluorescein) showed that BBB disruption in several (mainly limbic) brain regions of animals occurs within 5–30 min after acute seizures induced by either PTZ, bicuculline, pyridoxine or hyperthermia [20,21]. Accumulating evidence supports the notion that following epileptogenic insults both in humans and experimental animals, increased BBB permeability is the rule, rather than the exception. Indeed, BBB impairment has been reported in traumatic brain injury (TBI), stroke, brain infections, seizures, and status epilepticus (SE) [20]. This led to the concept that epileptogenic brain insults, including seizures induce a non-specific “opening” of the BBB that may allow increased drug penetration into the brain [22,23,24]. However, accumulating evidence indicates that changes in microvascular permeability following brain insults (including seizures), represent a relatively specific modulation of barrier functions, resulting in the transport of high molecular weight proteins (e.g., albumin) but not necessarily free permeability of the BBB to small ions such as potassium and protons [25]. Furthermore, experimental data indicate that this modulation of BBB function may be involved in complex molecular and cellular alterations that underlie the generation of seizures and epilepsy. In the following, we will review structural, molecular, and functional alterations in BBB integrity in response to seizures and brain injury and critically discuss the pathophysiological impact of such alterations.

## 3. BBB Structural Alterations in Epilepsy

Cellular components within the CNS are not fixed in a post-mitotic state but rather respond to numerous pathological and rheological signals to induce angiogenesis, leading to the formation of new microvessels and vascular remodeling [26]. Aberrant cerebrovascular angiogenesis has been found in the epileptic foci of patients with drug-resistant temporal lobe epilepsy (TLE) and in experimental models of TLE [26]. The aberrant angiogenesis was associated with loss of tight junctions and increased permeability to immunoglobulins G [27]. Electron microscopy studies showed increased pinocytotic activity, tight junction aberration and a thickening of the basement membrane in epileptic brains [28,29]. In addition to pinocytosis, pericyte-microglia clustering was observed in the epileptic brain, and likely contributes to BBB dysfunction [30,31]. Furthermore, astrocytes undergo morphological and functional alterations after insults and in the epileptic brain. These changes, including altered expression of K^+^ and water channels, seem to affect BBB function [32,33,34].

## 4. Why Do Epileptogenic Brain Insults and Seizures Alter the Morphology and Functionality of the BBB?

**Figure 2:**
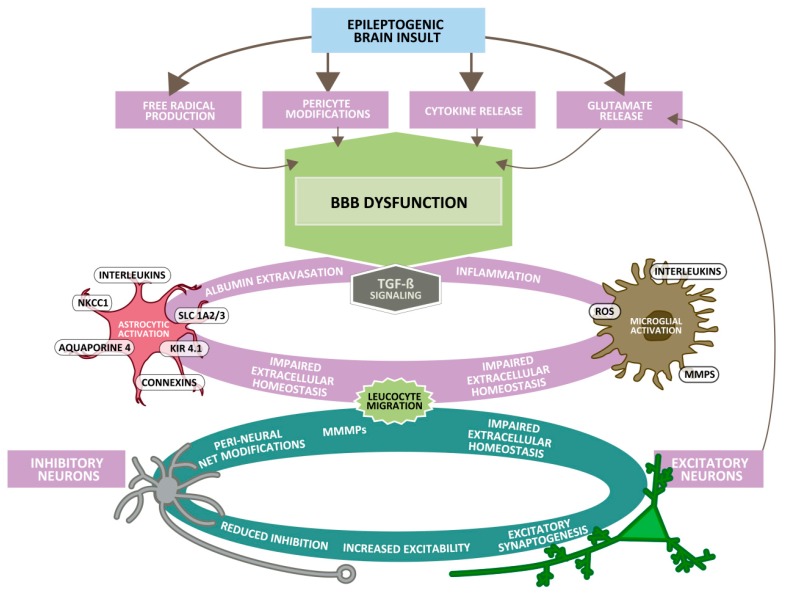
Underlying neurological complications after epileptogenic brain insults such as traumatic brain injury (TBI), stroke or encephalitis. The initial brain injury (top) is followed by the release of pro-inflammatory mediators (cytokines), production of free radicals, release of glutamate and pericyte modifications, which successively lead to dysfunctional blood–brain barrier (BBB) permeability with extravasation of serum-components (e.g., albumin). As a consequence, resident glial cells become activated (involving TGF-β signaling) and blood-borne leucocytes migrate into the brain parenchyma, which leads to cerebrovascular inflammation and disturbed extracellular homeostasis (e.g., due to increased movement of ions across the BBB or altered astrocytic potassium buffering via Kir 4.1 channels). As a consequence, the threshold for spreading depolarization decreases, neurons become hyperexcitable and seizures may occur. Altered glia function and neuronal excitability are further associated with synaptogenesis and neuroplasticity that may eventually lead to epilepsy, cognitive decline, and behavioral abnormalities. Cerebral edema and hemorrhagic transformation on the other hand are acute, direct consequences of increased BBB permeability. Abbreviations: Kir 4.1, inward-rectifying potassium channel; MMP, matrix metalloproteinase; NKCC1, sodium-potassium-chloride symporter; ROS, reactive oxygen species; Slc1, solute carrier family 1. Modified from Schoknecht et al. [41].

The mechanisms underlying seizure-induced BBB alterations are not fully understood. The relatively rapid increase in permeability during seizures (within ~30 min), together with a more lasting effect (for hours) [35] suggest multiple mechanisms act in concert to alter BBB properties. For example, seizure-induced increases in brain glutamate levels may lead to barrier dysfunction [21,36]. A recent study suggests that glutamate released during seizures increases the expression and activity levels of matrix metalloproteinases (MMP-2 and MMP-9) at the BBB, contributing to barrier dysfunction [37]. MMPs likely affect barrier integrity by digesting and remodeling the extracellular matrix surrounding brain capillaries and by degrading tight junction proteins that seal the endothelium [38,39]. Induction of seizures was also shown to induce inward current in pericytes leading to altered pericytic functions and BBB properties in vitro and in vivo [40]. As illustrated in Figure 2, glutamate release, reactive oxygen species (ROS), MMPs, angiogenic factors, inflammatory cytokines, autoantibodies, leukocyte adhesion and immune cell extravasation have all been discussed in this regard [5,40]. A leaky barrier contributes to seizure genesis through a positive feedback loop, in which seizures drive barrier leakage leading to more seizures, thereby promoting epilepsy progression. Thus, barrier leakage is both a consequence and a trigger of seizures and epilepsy, which will be discussed in detail below.

## 5. BBB Dysfunction May Alter Brain Uptake of Xenobiotics and Albumin

As described above, early studies with hydrophilic dyes (e.g., Evans blue) on seizure-induced impairment of BBB function indicated an “opening” of the BBB that may allow various drugs to enter the brain [22,23,24]. This, however, may not be the case, as “leakiness” was in many cases described to be a specific modulation in BBB properties and restricted to increased brain uptake of highly protein-bound hydrophilic dyes (e.g., Evans blue), hydrophilic drugs or some contrast agents (e.g., gadolinium), which normally do not enter the brain [25,42]. Indeed, the increase of most (if not all) dyes in brain parenchyma is likely a consequence of the fact that such dyes are highly bound to plasma albumin and that prolonged seizures induce extravasation of albumin through a transcellular pathway and hence, increased extravasation of albumin-bound dyes such as Evans blue into the brain parenchyma. In contrast, the brain/plasma ratio of small molecules not bound to plasma albumin is most often not changed or even decreased [10,25]. Albumin extravasation has not only been observed in patients with TLE (and animal models of TLE), but also in focal brain lesions associated with drug resistant epilepsy such as in focal cortical dysplasia, tuberous sclerosis complex, gangliogliomas and vascular malformations [20]. Tight junctions are only disrupted by very severe brain insults, so terms such as “BBB opening”, “BBB damage” or “BBB breakdown” in response to seizures are generally misleading. The observed “leakiness” more likely reflects modulation of endothelial properties and involves, at least in part, enhanced micropinocytosis while tight junctions remain intact [10,42]. Surprisingly, only relatively few studies examined whether prolonged, severe seizures alter the BBB permeability to commonly used drugs, such as ASDs. Assuming that albumin-bound drugs may penetrate through a dysfunctional BBB, it is important to note that the extent of plasma protein binding of ASDs largely differs, with some drugs (e.g., phenytoin, valproate, benzodiazepines) being highly (>90%) bound to plasma albumin, whereas other ASDs are hardly bound (e.g., ethosuximide, gabapentin, levetiracetam).

In a study performed by Potschka et al. [43], prompted by earlier observations that extracellular (unbound) brain levels of the highly-protein bound ASD phenytoin are lower in limbic areas of kindled than non-kindled rats [44], microdialysis experiments were made in amygdala-kindled rats and electrode-implanted, sham-kindled rats. The microdialysis probe was located directly adjacent to the stimulation/recording depth electrode in the basolateral amygdala (BLA)—the epileptogenic focus in the amygdala kindling model of TLE. Penetration of phenytoin to the extracellular fluid in the focus region (the stimulated BLA) was studied at different time points during and after seizure activity elicited in kindled rats. Access of phenytoin to the kindled focus was comparable in kindled rats two hours or fourteen days following a single generalized seizure compared to sham controls. When a single generalized seizure was elicited l0 min after phenytoin administration, average phenytoin levels in brain dialysates were lower (up to 44%) than those of sham controls. During self-sustained SE, which was induced by 30 min of electrical stimulation of the amygdala and lasted ~3 h, phenytoin access to stimulated amygdala tended to be lower early after drug administration, but reached control levels two hours later. BBB impairment was shown by enhanced brain uptake of Evans blue. These data clearly demonstrate that seizure-induced alterations in BBB function are not associated with increased brain levels of phenytoin, but rather decrease the free (non-protein bound) concentration of phenytoin in the extracellular compartment.

Marchi et al. [45] studied the effects of impaired BBB integrity on brain distribution of hydrophilic (deoxyglucose and sucrose) and lipophilic, highly protein-bound (phenytoin and diazepam) molecules. They tested the hypothesis that hydrophilic and lipophilic drug distribution is differentially affected by a dysfunctional BBB. BBB disruption was induced in rats by intracarotid injection of hyperosmotic mannitol. Drugs were determined and correlated with brain water content and protein extravasation. BBB disruption led to extravasation of serum albumin and radiolabeled drugs. The increase in total drug permeability was higher for lipophilic than hydrophilic compounds. However, BBB disruption markedly decreased the concentration of free phenytoin in the brain, which is comparable to our microdialysis findings with phenytoin after seizure-induced BBB disruption [43]. Marchi et al. [45] concluded that after BBB disruption, drug binding to protein is the key controller of total brain drug accumulation. Osmotic BBB disruption increased serum protein extravasation and reduced free phenytoin brain levels. These results are in line with in vitro recordings showing that imitating a “leaky” BBB by adding albumin to the perfusate, leads to “pharmacoresistance” of seizures to protein-bound ASDs (e.g., phenytoin, valproate) [46]. These studies suggest that in the context of epilepsy, free concentration of protein-bound ASDs are reduced in the presence of BBB dysfunction, which is in contrast to the often suggested notion that brain penetration of various drugs increases. Indeed, BBB “opening” to large molecules such as albumin does not necessarily imply free diffusion of small molecules or ions [25]. In line with this concept, analysis of various ASDs in brain extracellular fluid, brain tissue, cerebrospinal fluid and serum of patients with intractable epilepsy did not indicate any increase in free (protein-unbound, functionally relevant) brain ASD levels as a result of BBB impairment [47]. Duncan and Todd [23] already argued in 1991 that the state of the BBB should have little effect on brain tissue levels of ASDs because these are highly lipid soluble in contrast to hydrophilic dyes and thus hardly affected by BBB impairment, which is substantiated by our experimental and clinical data [43,47].

Under some conditions, however, BBB impairment may result in entry and accumulation of toxic bloodborne molecules including fibrinogen, hemoglobin, thrombin, iron-containing hemosiderin, free iron, plasmin (an extracellular matrix degrading enzyme) and environmental toxins [48]. Furthermore, magnetic resonance (MR) contrast agents containing the heavy metal gadolinium are used in association with MR imaging (MRI) in routine clinical practice to detect and quantify BBB leakage [48,49]. Under normal circumstances, gadolinium-based contrast agents, such as gadolinium-diethylenetriamine pentetic acid (Gd-DTPA), do not cross the intact BBB. This may either be a result of their physicochemical (hydrophilic or polar) properties or binding to plasma albumin [25,50]. However, gadolinium-based contrast agents may extravasate from the blood into the brain tissue even when the BBB is only partially compromised [51]. A recent study using a gadolinium-based contrast agent (gadobutrol) in patients with chronic epilepsy provided evidence for the occurrence of a BBB dysfunction, which is at least partly temporally and anatomically associated with epileptic seizures [35]. However, imaging data have shown region-specific accumulation of gadolinium even among patients with grossly normal brain tissue who underwent repeated injections of the contrast agent (4–18), suggesting that current thinking with regard to the permeability of the BBB is greatly oversimplified [51].

## 6. Increase in BBB Permeability Does Not always Lead to Seizures

Notably, under conditions in which the function of tight junctions is impaired, BBB dysfunction can result in significant changes in the concentration of ions, amino acid transmitters, proteins and metabolic products within the neuropil, contributing to abnormal neuronal activity, but not necessarily to seizures [5]. For example, claudin-5 is a tight junction protein expressed in endothelial cells (Figure 1) and is key for BBB functions. Interestingly, schizophrenia occurs in one third of individuals with 22q11 deletion syndrome (22q11DS), a population that is haploinsufficient for the claudin-5 gene [52]. In the mouse brain, adeno-associated virus-mediated suppression of claudin-5 results in localized BBB leakiness and a neurological phenotype with impairments in learning and memory, anxiety-like behavior, and sensorimotor gating [52]. A few weeks after claudin-5 suppression, mice also developed seizures. These results stress the notion that increased BBB permeability does not always lead to immediate seizures [20,53]. This is further supported by the fact that BBB malfunction has been recorded in various other neurological disorders (e.g., multiple sclerosis, Alzheimer’s disease, and cerebral ischemia), often without clear evidence for seizures. The reasons for the lack of seizures under these conditions may be related to the extent and specific nature of permeability increase, the affected brain networks (see [54]) or due to under-diagnosis of focal remote seizures, as was reported in patients with Alzheimer’s disease [55]. Furthermore, epilepsy is not a disease with a single cause, and apart from BBB dysfunction, there are multiple other brain alterations that cause seizures and epilepsy [56].

## 7. The Role of the BBB in Drug Resistance in Epilepsy

Drugs required to act in the brain, including ASDs, have generally been made lipophilic, and are thus able to cross the brain endothelium via the lipid biomembranes. However, such lipophilic drugs are potential substrates for efflux carriers of the BBB, particularly Pgp, which is predominantly located on the endothelial luminal membrane of the BBB. It is assumed that up to 50% of drug candidates may be substrates for Pgp [57]. Accumulating evidence indicates that under conditions in which the BBB is disturbed, ‘second line of defense’ mechanisms in brain capillary endothelial cells and perivascular glia may be up-regulated, including increased expression and functionality of Pgp and other drug efflux transporters [15,16]. This up-regulation has been suggested to contribute to ASD resistance in epilepsy, which affects about 30% of all patients and is a major problem in epilepsy therapy [58].

**Figure 3:**
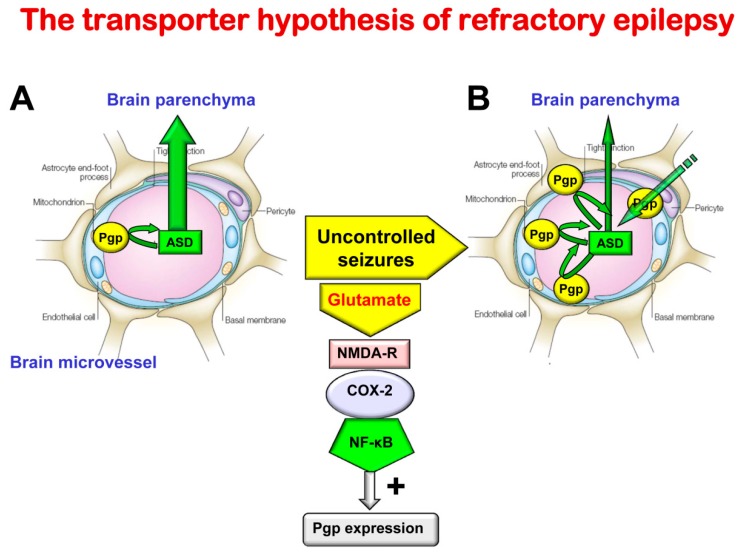
Illustration of the transporter hypothesis of drug-resistant (refractory) epilepsy. About 30% of all patients with epilepsy do not respond to current ASDs and are thus drug resistant [58]. Drug resistance is associated with increased morbidity and mortality [65]. Thus, understanding the mechanisms of drug resistance is important for developing more effective therapies. The transporter hypothesis is one of several hypotheses to explain why patients do not adequately respond to treatment with ASDs. Based on the transporter hypothesis, patients with drug resistant epilepsy have an increased expression of efflux transporters at the BBB in affected brain regions, leading to reduced penetration of ASDs. Indeed, preclinical experiments have shown that sustained seizure activity leads to induction of efflux transporters such as P-glycoprotein (Pgp) at the apical (luminal) membrane of brain capillary endothelial cells that form the BBB [15,16,20]. As shown in (**A**), in the absence of seizures (in the nonepileptic brain) ASDs easily penetrate through the BBB by diffusion, because they are small, lipophilic, non-ionized and only relatively weak substrates of Pgp [10]. For instance, based on in vivo data with chemical knockout of Pgp in rats and genetic knockout in mice, it has been calculated that the normal, constitutive expression of Pgp at the BBB restricts brain penetration of the ASD phenytoin by about 40–50% [10]. As shown in (**B**), following seizures, Pgp is overexpressed in the endothelial cells, so the ASD fraction bound to Pgp in the endothelial cells increases and the drugs are transported back into the blood, thereby reducing brain levels of these drugs in affected brain regions. For instance, for phenytoin it has been shown in vivo that Pgp can affect up to about 70–80% of brain drug uptake under these conditions [10]. In addition, ASDs that are already in the brain parenchyma undergo enhanced efflux from the brain, which is mediated by Pgp. This cannot be simply counteracted by increasing the dose, because Pgp is only overexpressed in epileptogenic or focal brain tissue, so increasing the dose would lead to toxic brain levels in other regions. By inhibiting Pgp (e.g., with tariquidar), the increased ASD efflux and the associated drug resistance can be reversed in preclinical animal models of epilepsy, and, anecdotally, in patients with drug resistant seizures [10]. The mechanisms underlying the Pgp increase in response to seizures [61] are also illustrated. The increase in Pgp resulting from enhanced glutamate release in response to seizures can be inhibited by NMDA receptor antagonists or COX-2 inhibitors [10]. It is important to note that it is not likely that drug resistance is mainly due to one mechanism such as the mechanism illustrated here, but rather several resistance mechanisms have been postulated which may even occur in concert in the same patient [60,66].

Tishler et al. [59] were the first to describe an increased expression of MDR1 in resected brain samples of patients with drug-resistant seizures and suggested that this overexpression may restrict brain accumulation of ASDs such as phenytoin, leading to the “transporter hypothesis” of refractory epilepsy (Figure 3) [15,16]. There is now a large body of evidence that multidrug transporters such as Pgp, BCRP, and multidrug resistance proteins (MRPs, ABCCs; e.g., MRP1, MRP2, MRP5), and their genes are over-expressed in capillary endothelial cells and astrocytes in human epileptic tissue that has been surgically resected from patients with medically intractable epilepsy [60]. The expression of multidrug transporters in astroglial end-feet might represent a “second barrier” under these conditions. The increased expression of Pgp induced by frequent seizures is thought to be a result of a complex signaling cascade (Figure 2), including seizure-induced glutamate release, which via stimulation of NMDA receptors leads to an induction of cyclooxygenase-2 (COX-2), which then, via NF-κB, increases the expression of Pgp [61]. In line with this hypothesis, the seizure-induced Pgp up-regulation is inhibited by NMDA receptor antagonists and COX-2 inhibitors [62,63,64].

In line with the transporter hypothesis, animal experiments have shown that (1) rats with spontaneous recurrent seizures not responding to ASDs (“ASD non-responders”) exhibit higher expression of Pgp at the BBB than “ASD responders”; (2) various ASDs are transported by rodent Pgp; (3) overexpression of Pgp is associated with lower brain levels of ASDs; and (4) the selective Pgp inhibitor tariquidar counteracts resistance to ASDs in a rat model of TLE [10]. Indeed, by using sensitive transport assays, several widely used ASDs have been shown to be substrates for both rodent and human Pgp and other drug efflux transporters [10,67].

The transporter hypothesis has also been investigated clinically. Using positron emission tomography (PET) and the PET ligand (and Pgp substrate) (*R*)-[^11^C] verapamil with and without the Pgp inhibitor tariquidar in 14 pharmacoresistant patients, eight seizure-free patients, and 13 healthy controls, Feldmann et al. [68] substantiated the association between regionally localized Pgp overactivity and pharmacoresistance in TLE, thus providing the first in vivo proof-of-concept of the transporter hypothesis in humans. In a large study on post-mortem brains from patients with drug-sensitive or drug-resistant chronic epilepsy and controls, Liu et al. [69] found highly localized overexpression of Pgp in the epileptogenic hippocampus of patients with drug-resistant epilepsy (on the vascular endothelium and end-feet of vascular glia, forming a ‘double cuff’) and concluded “our findings show that the expression of Pgp is compatible with the inherent assumptions of one current hypothesis of multidrug resistance”. Thus, pharmacologically overcoming Pgp overactivity could provide a potential treatment strategy, as demonstrated in animal models [10,70].

That such a strategy may be relevant in patients with epilepsy is suggested by several anecdotal reports on single patients with intractable epilepsy in whom the nonselective Pgp inhibitor verapamil was added to the ASD regimen [60]. One non-placebo-controlled open-label pilot study in 19 adult patients with refractory TLE found that co-administration of verapamil (120 mg daily in 13 patients and 240 mg daily in six patients) to the existing ASD treatment improved seizure control in a dose-dependent manner [71]. However, in a randomized, double-blinded placebo-controlled trial on verapamil (once daily 240 mg) as an add-on therapy in refractory epilepsy patients with focal onset seizures, no statistically significant decrease in seizure frequency was observed [72]. A more recent non-placebo-controlled open-label study, which investigated the efficacy of low-dose verapamil (20 mg three times daily) as adjunctive treatment in refractory epilepsy, reported that 10 out of 19 patients achieved 50% or more seizure reduction [73]. Clinical proof-of-concept trials with more selective Pgp inhibitors such as tariquidar or elacridar are needed, though the risks of such an approach need to be considered. Interest of pharmaceutical industry in developing selective Pgp inhibitors for add-on epilepsy therapy has declined after the failure of such Pgp inhibitors in several large cancer trials, which was mainly due to the unexpected toxicity of add-on treatment with Pgp inhibitors as a result of increased penetration and accumulation of cytotoxic chemotherapeutics in normal tissues [74].

The recently clarified signaling cascade that explains seizure-induced overexpression of Pgp (Figure 3) raises the possibility of direct manipulation of this overexpression (e.g., by inhibiting NMDA glutamate receptors or COX-2) [61,75]. Indeed, both NMDA receptor antagonists and COX-2 inhibitors, such as celecoxib, have been shown to prevent the seizure-induced increases in Pgp expression and functionality in rats. Celecoxib reversed ASD-resistance [75] extending previous results with the Pgp inhibitor tariqidar [70].

In addition to Pgp, some ASDs (e.g., lamotrigine) are transported by BCRP [76]. This may be important because BCRP is expressed at significantly higher levels at the human BBB than Pgp, whereas the opposite is true for the rodent BBB [77]. Uchida et al. [77] measured the BCRP monomer; however, because BCRP is a half-transporter that needs to homodimerize to be active, the BCRP quantity needs to be divided by two in order to estimate of the amount of functionally active BCRP. Furthermore, Uchida et al. [77] did not differentiate between transporter in the luminal membrane (that would presumably contribute to efflux transport) vs. transporter in vesicles (that would not contribute to efflux transport). Consequently, the relative functional activity of BCRP vs. Pgp at the human or rodent BBB is currently not known.

In contrast to Pgp and BCRP, most ASDs do not seem be transported by human MRPs, such as MRP1, MRP2, and MRP5, which are overexpressed at the BBB in drug-resistant epilepsy [78].

In addition to increased expression of efflux transporters, cytochrome P450 enzymes, known to be responsible for the metabolism of several ASDs enzymes (CYP3A4, CYP2C9, CYP2C19, CYP2A6 and CYP2E1), were demonstrated to be elevated in brain endothelial cells isolated from temporal lobe resections of drug-resistant epileptic subjects [17]. This indicates that increased metabolism of ASDs at the level of the BBB may add to drug resistance. Overall, these data suggest that alterations of the BBB in epilepsy decrease rather than increase drug penetration into brain parenchyma, thus arguing that BBB alterations in epilepsy are more complex than increased “leakiness”. 

## 8. Impairment of Barrier Functions and the Invasion of Inflammatory Cells into the Brain

Clinical and experimental evidence indicates that inflammatory processes contribute to the pathophysiology of several types of epilepsy [79]. However, the respective contribution of brain resident vs. brain invading (bloodborne) immune cells to epileptogenesis is not completely understood. Under healthy conditions, peripheral immune cells are restricted from CNS partly due to the BBB [80]. However, when BBB integrity is impaired, peripheral adaptive and innate immune cells, including monocytes, neutrophils, and different types of T cells and B cells, can enter the CNS, where they perform distinct cell-mediated effects which might be either neuroprotective, neurotoxic or both [80,81]. Indeed, trafficking of bloodborne immune cells through the BBB into the CNS represents a key process in neuroinflammation, consisting of a well-defined and regulated multistep cascade that involves consecutive adhesive interactions between leukocytes and the capillary endothelium. During the initial contact with the activated endothelium, leukocytes roll along the endothelium via a loose bond which is mediated by selectins. Subsequently, leukocytes are activated by chemokines presented on the luminal endothelial surface, resulting in the activation of leukocyte integrins and leukocyte arrest on the endothelium. After their firm adhesion, leukocytes use two transmigration processes to pass through the endothelial barrier, the transcellular route through the endothelial cell body or the paracellular route through the endothelial junctions and enter the perivascular space [82,83,84,85]. Within the perivascular space, the invading immune cells interact with other perivascular immune cells and are further guided towards the brain parenchyma by chemoattractants, such as chemokines and cytokines. Interestingly, before the multistep paradigm described above, immune cells may become activated in the periphery (e.g., in the lung) to reprogram their gene-expression profile, characterized by downregulation of their activation program and upregulation of cellular locomotion molecules together with chemokine and adhesion receptors [86].

Once invading leukocytes have crossed the glia limitans (the basement membrane laid down by astrocytes) by an enzyme-mediated process [87], they reach the brain parenchyma and are further activated to produce cytokines, resulting in massive immune cell recruitment and potentially a clinical disease [88]. This inflammatory response subsequently promotes changes in BBB functions from the luminal side (Figure 2). Astrocytic and microglial IL-1β and vascular endothelial growth factor (VEGF) have been suggested to promote increased BBB permeability [5]. Fingolimod, which impairs T cell migration to the CNS, was shown to exert antiseizure and antiepileptogenic effects in experimental TLE, indicating that brain invading T cells and cytokines released by these cells are involved in both ictogenesis and epileptogenesis [89].

There is evidence suggesting that leukocytes may also facilitate seizures and epileptogenesis from the luminal side of the vasculature, without entering the brain neuropil [90]. In a mouse model of pilocarpine-induced epilepsy, it was shown that the BBB endothelium exhibits an activated phenotype after a seizure and that increased Icam-1, Vcam-1, E-selectin and P-selectin expression promoted leukocyte rolling and arrest at the luminal surface of the vessels [90]. Remarkably, when leukocyte-endothelial interactions were inhibited, the number of recurrent seizures and the extent of BBB dysfunction were reduced. Furthermore, this approach prevented the development of epilepsy. These findings indicate that it may be possible to develop drugs that inhibit leukocyte-endothelial interactions in the periphery, thereby preventing disease initiation or progression within the brain and without the need to deliver drugs across the BBB.

In addition to T cells and neutrophils, peripheral monocytes are known to enter the brain after TBI and other epileptogenic brain insults and contribute to neuronal injury [91,92]. Indeed, brain invasion of peripheral monocytes has been recently implicated in epileptogenesis. Varvel et al. [93] demonstrated that infiltrating monocytes promote brain inflammation and exacerbate neuronal damage after SE in mice. Importantly, preventing monocyte recruitment reduced albumin extravasation and attenuated neuronal damage. We demonstrated that preventing monocyte recruitment prevents hippocampal neurodegeneration in a mouse model of viral encephalitis-induced epilepsy [94]. Brain resident cells such as microglia and astroglia contribute to the proinflammatory effects of brain invading immune cells [80,95].

## 9. Impairment of Barrier Functions and its Role in the Development of Epilepsy

In addition to immune cell migration through the impaired BBB, albumin extravasation has been suggested to play a role in epileptogenesis [96,97,98]. Indeed, 40 years ago, BBB disruption was proposed to cause acute seizures, as osmotic disruption of the BBB resulted in epileptic seizures in rats [99]. This finding was supported by a more recent study in which the BBB was transiently opened in naïve pig as well as in patients with brain tumors, leading to focal motor seizures that were related to albumin extravasation [100]. Albumin extravasation is found after several, if not all, epileptogenic brain insults, including TBI, stroke and SE [101] (Figure 2). Following extravasation, albumin is present in the brain parenchyma, but can also be taken up or bound to neurons, astrocytes and microglial cells. In astrocytes, albumin can be taken up via transforming growth factor beta (TGF-β) receptors. This is followed by a downregulation of inward rectifying potassium (Kir 4.1) and water (aquaporin 4; AQP4) channels, as well as glutamate transporters in these astrocytes (Figure 2). As a result, the buffering of extracellular potassium and glutamate is reduced, which facilitates NMDA receptor-mediated neuronal hyperexcitability and eventually induces epileptiform activity [33]. TGF-β signaling is further associated with transcriptional changes underlying inflammation, alterations in extracellular matrix (specifically perineuronal net around inhibitory interneurons) [102], excitatory synaptogenesis [103] and pathological plasticity [104], all considered important mechanisms that can contribute to lower seizure threshold during epileptogenesis [20,98,105]. The angiotensin II type 1 receptor antagonist, losartan, blocks brain TGF-β signaling and prevents epilepsy in different models of epileptogenesis [53,54,106]. Interestingly, a study using gadolinium-DPTA to assess BBB impairment in patients following TBI reported increased BBB permeability in 77% of patients with post-traumatic epilepsy compared with 33% of patients without epilepsy (*p* = 0.047) [107], suggesting a correlation between disrupted BBB and abnormal neuronal activity [108].

We recently explored the spatiotemporal evolution of extravasation of albumin and illuminated associated responses of the NVU contributing to early epileptogenic neuropathology [109]. For this purpose, we applied translational in vivo MRI (using gadolinium-DPTA as a contrast agent) and complementary immunohistochemical analyses in the rat pilocarpine model of TLE. A rapid BBB leakage was observed in epileptogenesis-associated brain regions that peaked between 1 and 2 d post-SE, and quickly declined thereafter, accompanied by cerebral edema generally following the same time course. At peak BBB leakage, serum albumin colocalized with NVU constituents, such as vascular components, neurons and glial cells. Surprisingly, astroglial markers did not colocalize with albumin. Furthermore, AQP4 was clearly reduced in areas of dysfunctional BBB, indicating a severe disturbance of astrocyte-mediated endothelial-neuronal coupling. In addition, a reorganization process of the NVU vasculature took place at sites of albumin presence, substantiated by reduced immunoreactivity of endothelial markers and changes in vascular basement membrane markers. Taken together, degenerative events at the level of the NVU, affecting vessels, astrocytes and neurons seem to outweigh reconstructive processes. Considering the rapidly occurring BBB leakage and subsequent impairment of the NVU, our data support the need for a rapid BBB-restoring treatment as a possible component of rational therapeutic intervention to prevent epileptogenesis and the development of other detrimental sequelae of epileptogenic brain insults such as SE. 

## 10. Overcoming the BBB in Epilepsy by Delivering Therapeutics to the Brain

As described above, BBB alterations in the epileptic brain may restrict brain entry of several major ASDs, which may add to the problem of drug resistance in epilepsy. There are various invasive and non-invasive strategies to bypass the BBB [110]; among those, local drug delivery is the strategy that has been most widely explored in epilepsy research [111,112,113,114]. ASDs or other neuroactive compounds may be either directly injected into the epileptic focus, for example, the hippocampus, or into the cerebrospinal fluid (CSF) by intracerebroventricular (i.c.v.) drug injection or may be administered subdurally. Numerous preclinical studies have demonstrated the effectiveness of such approaches. To our knowledge, the first proof-of-concept in patients was published by Madhavan et al. [115], who reported that subdural application of lidocaine-soaked gel foam adjacent to epileptogenic zones decreased spike counts in three patients with refractory focal epilepsy. More recently, the first clinical study on convection-enhanced delivery (CED) of the GABA_A_ receptor agonist muscimol into the seizure focus of patients with drug-resistant epilepsy was performed to investigate the safety and possible effectiveness of this approach [116]. CED is a drug-delivery technique that uses hydrostatic pressure to deliver a drug-containing fluid by bulk flow directly into the interstitial space within a localized brain region, thus achieving a wider distribution than conventional infusion [112]. Furthermore, recently, Cook et al. [117] reported that chronic i.c.v. administration of the ASD valproate is safe and effective in subjects with medically refractory epilepsy over many months. High CSF levels were achieved with corresponding low serum levels and this therapy was shown to be effective despite unsuccessful earlier use of oral valproate preparations.

Advantages of bypassing the BBB by subdural, intracerebral or i.c.v. drug administration include (1) overcoming BBB-associated resistance mechanisms such as overexpression of efflux transporters (e.g., Pgp), (2) allowing to use substances (e.g., peptides) that normally do not penetrate into the brain, (3) allowing to use toxins that are not suited for systemic administration, (4) achieving higher local (intracerebral) drug concentrations compared to systemic administration, and less adverse effects. However, there are also several disadvantages or problems with such approaches. (1) Intracerebral drug administration is an invasive method that is only applicable as an alternative to resective surgery in patients with pharmacoresistant epilepsy or patients with refractory SE. (2) Continuous drug administration (e.g., via minipump) is required for suppression of spontaneous recurrent seizures (over weeks, months, years or decades). (3) Because of diffusion barriers, drug distribution is restricted in target tissue, which is also true for i.c.v. injection, following which tissue distribution is only some millimeter at best, depending on the drug physicochemical properties such as lipophilicity and ionization at local pH. Nevertheless, local delivery strategies are an attractive option for treating neurological diseases since systemic side effects may be diminished and higher therapeutic doses may reach the brain, which may offer hope for many currently intractable patients for whom drug developments and surgical advances have proved disappointing [111,113]. Intracerebral drug delivery methods are already used routinely for brain tumor therapy and the increasing number of anecdotal reports on epilepsy therapy discussed above may indicate that they will be clinically developed for epilepsy soon. Which of the emerging techniques of BBB bypassing will prove the most appropriate for epilepsy therapy remains to be established.

## 11. Conclusions

The BBB is a highly selective, semipermeable interface regulating the passage of various molecules from the blood into the brain parenchyma and thereby playing a crucial role in maintaining strict neuronal homeostasis. The association between epilepsy and impairment of the BBB has long been suggested [118], leading to the concept that BBB dysfunction represents an important hallmark of the seizing brain. However, epilepsy research has faced a key question since then: whether BBB impairment is a cause or a consequence of epileptic seizures or perhaps both [119]. More recent data from brain imaging indicate that BBB dysfunction with albumin extravasation into brain parenchyma is the commonality of epileptogenic brain insults and may be suited as a biomarker of epileptogenesis in both animal models and patients [97,120]. Albumin extravasation into brain parenchyma can induce epileptogenesis and can sustain or even aggravate the epileptic condition (Figure 2). It is important to note that BBB dysfunction is most often locally restricted (focal) and transient. The consequence of seizure- or injury-induced BBB dysfunction for drug distribution into (and within) the brain is more complex because, as outlined in this review, associated upregulation of efflux transporters such as Pgp, BCRP and MRPs, may reduce functionally relevant free (unbound) drug concentrations in epileptogenic brain regions. Hopefully, a better understanding of the complex BBB alterations in response to seizures and epilepsy can lead to novel treatment strategies. A rapidly acting BBB-restoring treatment would be one component of rational therapeutic intervention to treat drug resistant seizures and prevent epileptogenesis and the development of other detrimental sequelae of brain injury [109]. Currently, the only applicable and most widely used therapeutic approach is to improve BBB integrity by treatment with glucocorticosteroids [5]. Indeed, the beneficial effect of add-on glucocorticosteroids for treatment of drug resistant seizures is associated with restoration of BBB function [121]. Similarly, the efficacy of natalizumab in drug-resistant epilepsy may be a result of preventing BBB–leukocyte interaction [121]. Other approaches for therapeutic BBB repair are currently under investigation, but in most cases translation of such approaches to the clinical arena is still far from reality. Further research in this field will hopefully lead to clinically usable treatment options for neurological complications associated with a dysfunctional BBB.
